# CD36 Signal Transduction in Metabolic Diseases: Novel Insights and Therapeutic Targeting

**DOI:** 10.3390/cells10071833

**Published:** 2021-07-20

**Authors:** Udayakumar Karunakaran, Suma Elumalai, Jun-Sung Moon, Kyu-Chang Won

**Affiliations:** 1 Innovative Center for Aging Research, Yeungnam University Medical Center, Daegu 42415, Korea; udayactech@gmail.com (U.K.); sumalakshmi@gmail.com (S.E.); 2 Yeungnam University College of Medicine, Daegu 42415, Korea

**Keywords:** CD36 antigens, diabetes mellitus, inflammation, insulin-secreting cells, oxidative stress, reactive oxygen species

## Abstract

The cluster of differentiation 36 (CD36) is a scavenger receptor present on various types of cells and has multiple biological functions that may be important in inflammation and in the pathogenesis of metabolic diseases, including diabetes. Here, we consider recent insights into how the CD36 response becomes deregulated under metabolic conditions, as well as the therapeutic benefits of CD36 inhibition, which may provide clues for developing strategies aimed at the treatment or prevention of diabetes associated with metabolic diseases. To facilitate this process further, it is important to pinpoint regulatory mechanisms that are relevant under physiological and pathological conditions. In particular, understanding the mechanisms involved in dictating specific CD36 downstream cellular outcomes will aid in the discovery of potent compounds that target specific CD36 downstream signaling cascades.

## 1. Introduction

CD36 is a transmembrane glycoprotein found in platelets, mononuclear phagocytes, adipocytes, hepatocytes, myocytes, taste bud cells, and a variety of other cell types. It plays a role in lipid accumulation, inflammatory signaling, energy reprogramming, oxidative stress, and apoptosis, all of which contribute to metabolic dysfunction [1,2,3,4,5]. Many physiological and pathological factors, such as long-chain fatty acids and proteins containing thrombospondin structural homology domains and oxidized phospholipids, including oxidized LDL (oxLDL), perturb CD36 function and induce metabolic diseases [6]. The scavenger receptor functions of CD36 have been widely researched due to the possibility of CD36 ligands in disease etiology. For example, higher sCD36, a non-cell-bound CD36 found in human plasma that indirectly reflects CD36 expression in tissues, has been linked to obesity, insulin resistance, and diabetes according to recent community-based cohort research [7,8,9]. Yang et al. also found that inhibiting the integral membrane protein CD36 with pharmacological agents lowers body weight growth and improves glucose tolerance [10]. Furthermore, a rise in CD36 levels contributes to the advancement of obesity-related metabolic dysfunctions by increasing lipid accumulation and inflammation. These studies also showed that CD36 is a critical player in the development of obesity and type 2 diabetes caused by a high-fat diet [11]. Despite the fact that the pathophysiology of these metabolic illnesses is complicated, there is evidence that CD36 is implicated in the abnormal signaling and tissue damage observed in this context. Since CD36 is expressed in a variety of tissues, it is impossible to discuss every aspect within the scope of this review. In this review, we look at the molecular links between CD36 and metabolic disease in a broader framework, focusing on pancreatic β-cells and other cell systems during the progression of metabolic disorders.

## 2. Comment on CD36-Assisted Fatty Acid Uptake

The cellular fatty acid uptake rate is determined by the presence of CD36 at the cell surface, which is regulated by subcellular vesicular recycling from endosomes to the sarcolemma (lipid raft). More specifically, VAMP2, a vesicle-associated membrane protein (VAMP) isoform, participates in CD36 translocation to the plasma membrane upon stimulation with insulin [12,13,14,15]. Upon removal of insulin, CD36 is rapidly internalized, leading to a concomitant decrease in the fatty acid uptake rate. Under normal conditions, the endosomal lumen is slightly acidic (pH ~5.5), which allows optimal endosomal retention of CD36 and concomitant low rates of fatty acid uptake under non-challenging conditions. Moreover, protein palmitoylation governs several steps of the insulin cascade and consequently contributes to the regulation of substrate transporter recycling between intracellular compartments and the plasma membrane. However, palmitoylation of VAMP2 might regulate the membrane localization of VAMP2, and the inhibition of palmitoylation has been shown to decrease glucose uptake in adipocytes [16]. Given the well-established relationship between palmitoylation and protein targeting of membranes, it is possible that the palmitoylation of VAMP2 enhances association with CD36, thus facilitating the arrangement of CD36 translocation to the cell surface. Most likely, lipid rafts/caveolae and their marker protein, caveolin-1, may play a crucial role in the post-translational stabilization of CD36 at the cell membrane surface. Caveolae represent a morphologically identifiable subset of lipid rafts, and caveolin-1 is essential for the formation of the characteristic flask-shaped invaginations of the plasma membrane [17]. The depletion of caveolin-1 or deletion of the caveolin-1 gene is known to result in the complete loss of caveolae and CD36 surface availability and thereby, the regulation of fatty acid uptake [18,19,20,21]. These results indicate the importance of intracellular signaling/trafficking events in CD36 function, as well as the metabolic fates of FAs following uptake. In addition to residing on the cell surface, CD36 localizes to intracellular vesicles and mitochondria, where it interacts with carnitine palmitoyl transferase 1, which is the key mitochondrial enzyme regulating FA transport into mitochondria and oxidation and is especially important for meeting increased metabolic demands during muscle contraction [22,23,24]. The detailed signaling pathways mediating CD36 trafficking are still unclear, but there is emerging evidence indicating a role for AMPK activation [25]. Independent of its physiological function, it is obvious that CD36 may participate in abnormal FA utilization and its deleterious consequences during insulin resistance and obesity (reviewed in Refs. [26,27,28,29,30,31]).

## 3. Structure and Post-Translational Modifications of CD36

**Figure 1:**
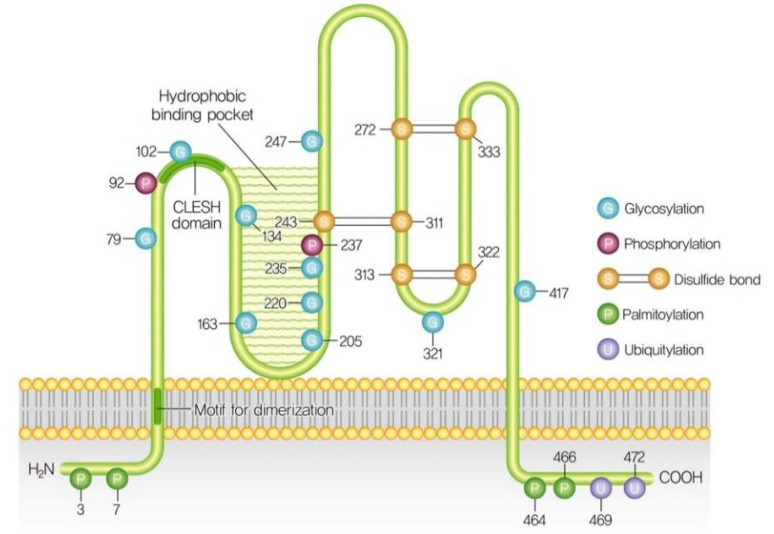
CD36 structure and post-translational modifications. (Adopted from Ref: [28], copyright 2020 © Korea Diabetes Association).

CD36 is also known as fatty acid translocase (FAT), glycoprotein IIIb (GPIIIb), or platelet glycoprotein IV, PAS4, and SR-B2 [32]. The human CD36 gene consists of 472 amino acids with a molecular weight of 53 kDa and is located on chromosome 7 (7q11.2) [33]. Analysis of the amino acid sequence of CD36 predicts a hairpin-like configuration with two transmembrane domains and two short cytoplasmic tails at the N- and C-termini. The extracellular loop contains a large hydrophobic cavity responsible for the recognition of ligand binding to moieties such as advanced glycated end products, cholesterol, and fatty acids [34,35,36]. The ligand-binding site also contains multiple glycosylation sites and three disulfide bridges essential for the intracellular processing and targeting of the protein to the outer leaflet of the plasma membrane [37]. In contrast, partially glycosylated CD36 mutants were also distributed to the cell surface without alterations in ligand binding [38]. However, the regulatory mechanism of CD36 glycosylation that leads to fatty acid absorption or fatty acid transport is not known. Contrary to glycosylation, myocellular fatty acid uptake is enhanced by the *O*-GlcNAcylation of CD36 via translocation to the sarcolemma [39]. *O*-GlcNAcylation is the dynamic regulatory post-translational modification of proteins stimulated by the addition of *O*-linked β-*N*-acetyl glucosamine (*O*-GlcNAc) to serine and threonine residues on target proteins [40,41]. However, increased *O*-GlcNAcylation levels of CD36 promote high-fat uptake by gastric cancer cells, which is needed for their metastasis [42]. Increased CD36-*O*-GlcNAcylation has also been linked to the reprogramming of cardiac fatty acid and glucose metabolism during acute or chronic stresses [43], although further studies are necessary to define the alteration of metabolic reprogramming by CD36 *O*-GlcNAcylation. CD36 is also palmitoylated and depalmitoylated by palmitoyl-transferases (PATs) and palmitoyl-protein thioesterases respectively. CD36, *N*-, and *C*-terminal tails contain cysteine residues that are palmitoylated by PATs, which anchors the protein to the plasma membrane in a reversible process. Under palmitic acid stimulation, CD36 is palmitoylated by PATs in the endoplasmic reticulum (ER) in a reversible thioester linkage between palmitate and cysteine residues [44,45]. However, the inhibition of CD36 palmitoylation causes CD36 ubiquitylation and degradation in the ER [46]. CD36 has two polyubiquitylation Lys469 and Lys472 sites in the C-terminus. However, CD36 ubiquitination does not affect CD36 distribution between cell surfaces from intracellular storage compartments [47]. Recently, CD36 was found to be a target of ubiquitin-specific peptidase 10 (USP10), which is a mammalian deubiquitinase. USP10 directly stabilizes CD36 via deubiquitination, and the inhibition of USP10 increases the polyubiquitylation of CD36 and enhances its proteasomal degradation, leading to reduced CD36-mediated fatty acid uptake by macrophages [48]. CD36 can also be monoubiquitinated by the E3 ubiquitin-protein ligase Parkin, which warrants further investigation [49]. Notably, CD36 is also phosphorylated at Thr92 by protein kinase C (PKC) and Ser237 by protein kinase A (PKA) within the extracellular loop, which is linked to the inhibition of fatty acid uptake [50,51]. Along these lines, published evidence suggests that CD36 has four acetylation sites (Lys52, Lys166, Lys231, and Lys403), and the functional effects of these acetylations are not yet known [52]. Sulfosuccinimidyl oleate (SSO), an irreversible inhibitor of CD36, binds to CD36 at Lys164 via the formation of *N*-hydroxysuccinimidyl esters in the fatty acid-binding pocket, leading to the inhibition of fatty acid intake and/or fatty acid-induced signaling [53]. Thus, further investigations are needed to know the role of CD36 acetylation at Lys52, Lys166, Lys231, and Lys403 and its downstream signaling. Taken together, CD36 could be targeted by different post-translational modifications in different tissues in a context-dependent manner and have been incompletely resolved, which could be a topic for future studies (Figure 1).

## 4. Role of CD36 in Pancreatic β-Cell Pathophysiology

### 4.1. Glucotoxicity

CD36 exerts fundamental biological functions at the cellular and tissue levels in multiple homeostatic and pathological processes by its distinct binding sites. Pancreatic β-cells play a central role in regulating glucose metabolism to sustain energy homeostasis by mediating a balance between insulin, an anabolic hormone, and glucagon, a catabolic hormone. Pancreatic β-cells require suitable sensors and signaling molecules that are integrated to modulate insulin secretion and maintain homeostasis. However, type 2 diabetes (T2D) is based on the inability of pancreatic β-cells to sustain a compensatory secretory response, leading to insulin secretory dysfunction and the pathogenesis of T2D. CD36 is the most generously expressed transporter among fatty acid transporters in human islets. It is located in the plasma membrane and co-localizes with insulin granules [54]. Interestingly, CD36 was shown to traffic between intracellular compartments and the cell surface in a vesicle-mediated process [55]. It has been well established that glucose potentiates fatty acid-induced β-cell death via apoptosis [56,57]. Similarly, the overexpression of CD36 in β-cells increases the uptake of fatty acids and leads to metabolic and functional dysfunction [58]. We also reported that glucotoxicity influences pancreatic β-cell dysfunction by increasing the influx of free fatty acids (FFAs) via CD36 [59]. To evaluate the mechanisms by which glucotoxicity affects β-cell dysfunction, we investigated CD36 expression and trafficking in β-cells and observed that Rac1, a small Rho family protein, displays increased glucose-mediated CD36 expression on the membrane surface in pancreatic β-cells [60]. The importance of Rac1 signaling in early-phase insulin secretion was previously demonstrated in β-cell-specific Rac1-deficient mice via the inhibition of F-actin depolymerization [61,62]. Glucose stimulates the recruitment of insulin granules to the cell membrane through actin remodeling, which is necessary for glucose-stimulated insulin secretion [63,64]. The F-actin function is coupled to SNARE-associated proteins such as syntaxin 1, syntaxin 4, and SNAP-25 in β-cells, and many F-actin-binding proteins interact with the SNARE machinery [63,65,66,67]. Several studies have shown that deficiencies in SNARE proteins are likely caused by high glucose and might contribute to cell dysfunction in disease states [68,69,70]. More considerations concerning SNARE protein function are discussed in the review article by Gaisano et al. [71]. Recently, it was shown that CD36 overexpression attenuates insulin secretion in human islets through the reduction of the exocytotic genes Snap25 and Vamp2, resulting in a decreased number of docked granules. It was further demonstrated that CD36 overexpression attenuates insulin signaling, resulting in the accumulation of the transcription factor FoxO1 in the nucleus as a potent transcriptional repressor of exocytotic genes. Interestingly, the inhibition of CD36 was shown to upregulate exocytotic gene expression in human islets, improving granule docking and resulting in increased insulin secretion without affecting insulin content [72]. Further research is required to identify the roles of CD36 in exocytotic gene function as well as whether F-actin function is involved in suppressing insulin secretion by CD36. Such studies will provide greater insights into the mechanisms of how CD36 induces metabolic dysfunction in pancreatic β-cells.

On the other hand, evidence suggests that hyperglycemia leads to the generation of reactive oxygen species (ROS), resulting in increased oxidative stress in β-cells [73,74]. The activation of Rac1 increases the production of oxidants, such as H_2_O_2_, via the activation of NADPH oxidase (NOX), which might trigger oxidative stress linked to β-cell death in T2D [75]. It was previously observed that CD36 deficiency reduces NOX activity and attenuates obesity-associated oxidative stress in the heart [76]. We also observed that Rac1 mediates NOX activity, leading to an increase in CD36 at the plasma membrane and that Rac1 and NOX inhibition can abrogate CD36 downstream signaling damage in response to high glucose [60]. However, it remains unknown how CD36 translocation to the plasma membrane is detected after Rac1-NOX activation by high glucose. One possible explanation may involve the palmitoylation of CD36 by supraphysiologic glucose levels. High-glucose-induced Rac1-palmitoylation has been suggested to be a driving force behind the activation of NOX, which in turn would alter the localization of Rac1 remodeling in diabetic retinopathy [77]. Nonetheless, a current topic of research is to elucidate how protein palmitoylation influences the function of proteins under high glucose conditions in pancreatic β-cells. It should be noted that increasing ROS production can alter cellular dysfunction stimulated by the activation of stress kinases by changing the balance of antioxidant enzymes. Previous findings also reported that CD36 altered cellular signaling under metabolic stress conditions by downregulating the redox-sensitive nuclear factor Nrf2 via Fyn kinase in murine vascular smooth muscle cells [78]. In addition, CD36 signaling in response to scavenger ligands leads to the activation of Src and MAPK family kinases, such as Lyn and c-Jun *N*-terminal kinase (JNK) in macrophages and platelets, whereas Fyn and p38 are the primary mediators of endothelial cells [79,80,81]. We also reported that Rac1-CD36 signaling by high-glucose-induced JNK and p38MAPK activation and the inhibition of CD36 inhibition blocks high-glucose-induced oxidative stress. Lots of evidence has suggested that ER stress is linked to insulin resistance, and pancreatic β-cell ER expansion was detected in patients with T2D [82,83]. Cells activate adaptive, self-protective mechanisms in response to ER stress, which are collectively referred to as the ER stress response (also named UPR). These include enhanced ER size, increased ER folding capacity through the manipulation of chaperones and foldases, decreased biosynthetic load, and the increased clearance of unfolded proteins through the stimulation of ER-related degradation. When these systems fail to alleviate the stress, apoptosis is triggered. Subsequent work from our lab has demonstrated that chronic glucose exposure or thapsigargin treatment induces ER stress through the reduced expression and activity of insulin and PDX1 with CD36 induction. Inhibition of CD36 in β-cells by metformin treatment or by using CD36 siRNA was shown to prevent the generation of ER stress markers and stress kinase activation [84]. It is clear that signaling related to CD36 regulation and its dynamics has impacts on oxidative stress, and understanding this linkage warrants further investigation. Given that CD36 signaling is related to numerous pathological events, β-cell CD36 downstream targets need to be further investigated.

### 4.2. Lipotoxicity

Hyperglycemia with elevated FFAs plays a significant role in insulin resistance and β-cell dysfunction in T2D [85,86,87]. Many studies have shown that glucose enhances fatty acid-induced β-cell death via apoptosis [56,57]. Fatty acid–glucose balance is essential for maintaining normal β-cell function, but lipotoxicity-induced β-cell dysfunction occurs with increased ROS, ceramide and nitric oxide levels, and mitochondrial perturbations [88,89]. Studies in Zucker diabetic fatty (ZDF) rats, an obesity-induced diabetic animal model, confirmed FFA-induced ceramide accumulation leading to β-cell apoptosis [90]. Another study demonstrated that superoxide production was elevated in islets isolated from Zucker lean fatty (ZLF) and Zucker diabetic fatty (ZDF) rats in the presence of glucose [91]. The resting superoxide content of ZDF rat islets was higher than that of Zucker lean control islets and was accompanied by the alteration of mitochondrial morphology. The FFA-induced formation of ceramide also induces the generation of ROS and DNA fragmentation [92]. Collectively, oxidative stress and mitochondrial dysfunction result in endogenous antioxidant impairment. However, plasma from patients with obesity and T2D shows enhanced levels of ceramides, which may serve as biomarkers for the diagnosis and treatment of obesity and diabetes [93,94,95]. Based on its role in FFA uptake, our lab showed that CD36 might promote ceramide-induced β-cell dysfunction by the Src-mediated tyrosine phosphorylation of Vav, a guanine nucleotide exchange factor (GEF), and also elevate metabolic pathways via its GEFs activity [96]. Evidence suggests that saturated fatty acids impair insulin secretion and induce insulin resistance via Src signaling in T2D [97]. Accordingly, we hypothesize that the induction of CD36 could be recapitulated in cells with functional vav tyrosine phosphorylation by Src promoting Rac1 signaling that generates ROS by NOX. Our results indicate that CD36-mediated Src-Vav activation is necessary for optimal Rac1-NADPH-induced superoxide production. To determine whether or not CD36 is linked to Vav-Rac1-NOX activation, we performed pharmacological inhibition of Src activity or CD36 siRNA, which significantly reduced ceramide-induced RAC1-NOX and inhibited ROS formation [98]. Holzer et al. demonstrated that saturated fatty acids stimulate stress-signaling activation by Src via transfer to a membrane micro domain [99]. In addition, the FFA-mediated Src-dependent Vav phosphorylation coordinates the engagement of Rac1-NOX-JNK signaling, which contributes to insulin resistance, obesity, and the production of inflammatory cytokines [100,101]. In podocytes, CD36-dependent uptake of palmitic acid leads to impaired mitochondrial energy metabolism, the alteration of mitochondrial and ER morphology, increased levels of mitochondrial ROS, the depolarization of mitochondria, ATP depletion, and apoptosis [102,103,104]. 

A recent study showed that p66Shc mediates lipotoxicity-induced impaired metabolic changes that promote pancreatic β-cell dysfunction and apoptosis in diabetes [74]. Earlier studies have suggested that p66Shc serine36 phosphorylation by JNK leads to ROS production and cell death [105]. Activated JNK combined with p66Shc serine36 phosphorylation activation to induce mitochondrial ROS in response to CD36 signaling promote cellular dysfunction. Cells lacking this pathway, as a consequence of CD36 inhibition, significantly block ceramide-induced β-cell dysfunction. Evidence points to CD36 signaling-generated H_2_O_2_, which promotes cysteine sulfenylation, a post-translational modification important to the augmentation of platelet activation and aggregation [106]. Peroxiredoxins, a thioredoxin-dependent peroxide reductase family of antioxidant proteins, catalyze the reduction of both hydrogen peroxide and alkyl peroxides to water and their corresponding alcohols [107,108]. The expression of peroxiredoxin-3 (PRDX3) is restricted to β-cells in pancreatic tissue. The oxidation of peroxidase cysteine to sulfonic acid (peroxiredoxin-SO3) promotes the accumulation of oxidized PRDX3 in mitochondria, which favors mitochondrial permeability transition pore (MPTP) opening and mitochondrial swelling [109]. Importantly, ceramide-induced sulfenylation is reduced in the presence of CD36 inhibition, which is consistent with a CD36-dependent mechanism. However, reduced PRDX3 is regulated by the thioredoxin–thioredoxin reductase system. Accordingly, we hypothesize that the inhibition of thioredoxin could be recapitulated in cells with functional thioredoxin-interacting protein (TXNIP) by preventing peroxiredoxin-3 activity in response to ceramide. We observed that TXNIP translocates to mitochondria and inhibits the antioxidative protein thioredoxin in response to ceramide. Moreover, ceramide-induced nuclear factor kappa-light-chain-enhancer of activated B cells (NF-κB) activation has been shown to increase TXNIP expression in β-cells [110]. This finding suggests that CD36 plays an important role in the initiation of oxidative stress induced by ceramide under conditions of β-cell failure. Thus, exploration of CD36 warrants further investigation.

Under normal conditions, mitochondria in β-cells persistently undergo fusion and fission. These processes may function to refute the negative impacts of the long-term presentation of β-cells to palmitate under high glucose conditions, causing mitochondrial fragmentation and impeding network dynamics by abolishing fusion and fission activity [111]. There are two powerfully contradictory processes that determine mitochondrial shape and morphology: fusion and fission. The ablation of both fusion and fission produces a significant effect on the progression of cells to apoptosis [112]. It has been reported that the mitochondria of β-cells from Zucker diabetic rats are divided, suggesting an imbalance in the mitochondrial fusion and fission process [91]. The exposure of β-cells to high-fat glucose conditions causes the discharge of Ca^2+^ from the ER to the cytoplasm, driving a rise in the cytosolic Ca^2+^ concentration that reflects expanded mitochondrial Ca^2+^ uptake. Increased mitochondrial Ca^2+^ uptake improves local buffering capacity and the discharge of proteins competent in apoptosis induction. Hence, Ca^2+^ activates the phosphatase calcineurin, which dephosphorylates and inactivates dynamin-related protein 1 (Drp1), a master controller of mitochondrial fission [113]. It can be assumed that crosstalk between the ER and mitochondria may promote cellular commitment to apoptosis through Ca^2+^. Recently, the presence of inositol trisphosphate receptor (IP3Rs) has been implicated in proapoptotic Ca^2+^ transfer between the ER and mitochondria [114]. An important remark is that Akt restrains the ER-to-mitochondria Ca^2+^ exchange by means of IP3R3 and ensures against Ca^2+^ intervened apoptosis [115]. Interestingly, CD36 was found to be overexpressed in obese diabetic islets and suppressed the insulin-signaling PI3K/AKT pathway, as well as its downstream transcription factors [72]. These effects result in ER-mitochondrial reprogramming, which contributes to the development of β-cell death and failure. The different pathways enacted by CD36 require further confirmation of the precise roles of CD36 signaling pathways in β-cell failure.

### 4.3. OX-LDL and Amyloid Deposition 

CD36 can generate cell-specific reactions to multiple ligands through the binding of context-specific binding partners that contribute to the development of β-cell dysfunction. As described above, it has been shown that ER stress is connected to insulin resistance in diabetes, and, conjointly, an expansion of ER was recognized in β-cells from patients with T2D [81,82]. Oxidized-LDL (oxLDL)-induced ER stress activation is coupled with oxidative stress, leading to β-cell dysfunction and death [116]. It has been shown that oxLDL induces β-cell dysfunction and apoptosis via the activation of ROS and that radical lipid hydroperoxides contribute to JNK activation [117,118,119]. However, the downstream mechanism by which JNK leads to apoptosis is not yet clear, and the crosslink between oxLDL and CD36 may promote cellular commitment to apoptosis through JNK enactment. A previous study reported that oxLDL intervenes with the JNK-dependent phosphorylation of p66Shc in endothelial cells, which contributes to oxidative stress and the atherogenic progression [120], Thus, we cannot preclude a role for PRDX3 oxidation in CD36 signaling. In this way, p66Shc can result in the overproduction of H_2_O_2_, which in turn can react with PRDX3 to cause toxic mitochondrial dysfunction and apoptosis. 

Regarding the molecular mechanisms involved, CD36 overexpression partners with the increased uptake of oxLDL without exerting additive effects on oxLDL toxicity [121]. Evidence suggests that CD36 causes a mitochondrial metabolic switch from oxidative phosphorylation to superoxide generation in reaction to oxLDL, which subsequently promotes NF-κB activation and the generation of pro-inflammatory cytokines [122]. Hence, redox status is subordinate on the degree to which a cell’s components exist in an oxidative state, whereby a reducing environment inside cells can prevent oxidative stress. oxLDL also initiated the ASM/ceramide signaling pathway, which is involved in macrophage apoptosis via the ER stress pathway [123]. However, the downstream targets of oxLDL in β-cells are not well known, and additional studies are needed. 

On the other hand, β-cells have a lower abundance of antioxidant defense enzymes, such as superoxide dismutase (SOD), catalase, and glutathione peroxidase (GPx) [124,125,126]. As such, the administration of antioxidant supplements can increase the defense capacity of islet cells to cope with oxidative stress [127]. Vitamin E is a redox-active natural compound that downregulates levels of ROS under different experimental conditions [128,129,130,131]. Interestingly, vitamin E reduces the uptake of OX-LDL by inhibiting CD36 expression via the PPARγ signaling pathway [132,133,134]. Furthermore, vitamin E facilitates the activation of PI3Kγ/AKT, leading to increased VEGF expression as well as elevation of cell survival and angiogenesis via its ability to increase tissue remodeling [135]. In addition, genetic data indicate that VEGF is a major regulator of islet vascularization and the revascularization of transplanted islets, and reduced beta-cell VEGF expression impairs glucose-stimulated insulin secretion [136]. Furthermore, the addition of vitamin E induces insulin secretion and islet-cell survival and functionality by enhancing PDX1, a master regulator of insulin gene expression [137,138]. Thus, the elevated expression of CD36 in beta cells exposed to elevated OX-LDL results in increased ROS expression that could induce discrete oxidative stress. Therefore, we suggest that the vitamin E-induced elevation of insulin expression may be mediated by CD36 inhibition, which may explain, at least in part, the reported protection against oxidative stress. Further studies are needed to elucidate the relationship between CD36, vitamin E, and OX-LDL in the pancreatic β-cell dysfunction.

**Figure 2:**
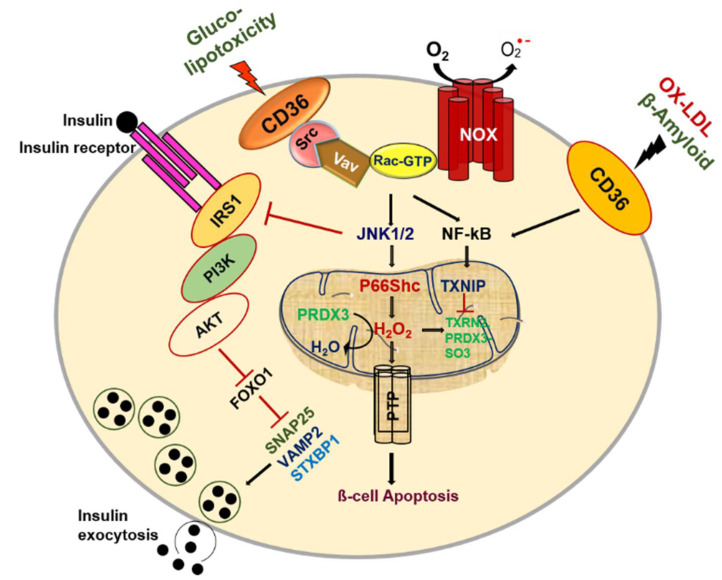
CD36 signal transduction in pancreatic β-cell dysfunction.

Among the variety of proapoptotic factors present in pancreatic β-cells, islet amyloid polypeptide (IAPP) is thought to play a crucial role in β-cell apoptosis. The proapoptotic effects of IAPP are mediated through a complex sequence of signaling events that lead to defects in mitochondrial dysfunction, autophagy, local inflammation, oxidative stress, cytokine production, and the enactment of signaling pathways driving to apoptosis [139,140,141,142]. Interestingly, CD36 can create a solid pro-inflammatory reaction through its interaction with secreted amyloid-beta 1–42 (Aβ) in macrophages [143]. However, the presence of this CD36-dependent pro-inflammatory signaling hub within the pancreatic β-cells has not been studied and warrants further investigation (Figure 2).

## 5. Role of CD36 in Peripheral Insulin Resistance and Metainflammation

Insulin resistance could be a trademark of metabolic defects, and it paves the way for the development of T2D. Impaired glucose tolerance, alcoholism, smoking, hypercholesterolemia, hypertriglyceridemia, low HDL, and hypertension are some of the risk factors involved in insulin resistance [144,145]. Modifications of lipid metabolism upon chronic FA oversupply interceded by CD36 lead to the accumulation of specific lipid species that are especially critical for the development of insulin resistance [146,147]. Increasing evidence shows that dysfunctional adipose tissue is associated with insulin resistance. This mechanism could be related to the recruitment of macrophages and other immune cells, which aggravate systemic inflammation and ectopic fat accumulation [148,149,150]. Accumulating evidence suggests that insulin resistance is not only induced by fat accumulation in adipose tissues but also by pro-inflammation caused by ectopic fat toxic lipids, such as ceramides, which alter the insulin signaling pathway, generates ROS-induced ER stress, and induces inflammation during the development of T2D [151]. It has also been reported that CD36-dependent inflammation and the apoptosis of adipocytes in response to diet-induced obesity reduces insulin sensitivity [152]. These changes are related to the activation of stress signaling pathways, such as the JNK, NF-κB, and ER stress cascades [153,154]. Moreover, CD36-associated JNK activation correlated with the impaired tyrosine phosphorylation of IRS-1, and IRS-2 blocks interaction with phosphoinositide 3-kinase (PI3K), thereby inducing insulin resistance. A recent study by Vandanmagsar et al. [155] detailed that lipotoxicity-associated increments in intracellular ceramide induce caspase-1 cleavage in macrophages and fat tissue through nucleotide-binding domain leucine-rich repeat (NLR) and pyrin domain-containing protein 3 (NLRP3) inflammasome activation. The development of insulin resistance and T2D has been connected with the expanded release of proinflammatory cytokines, which may impair insulin signaling, and this is supported by a clinical study in which T2D elevated FFAs and IL-1β in plasma, promoting insulin resistance [156]. Other evidence suggests that NOX-TXNIP-dependent ROS production drives caspase1-dependent IL-1β secretion by NLRP3 activators [157,158]. We also showed that CD36 signaling leads to ROS production due to TXNIP translocation to the mitochondria and represses the antioxidative protein thioredoxin in reaction to ceramide [110]. Indeed, TXNIP plays a role in producing IL-1β through NLRP3 inflammasome enactment beneath ER stress within pancreatic β-cells [159]. Inflammasome activation impedes insulin signaling in several target tissues to diminish glucose tolerance and insulin sensitivity [160]. Likewise, it has been reported that CD36-mediated pathogenic inflammasome activation is linked to cholesterol accumulation in the inflammatory process of atherosclerosis [161]. 

**Figure 3:**
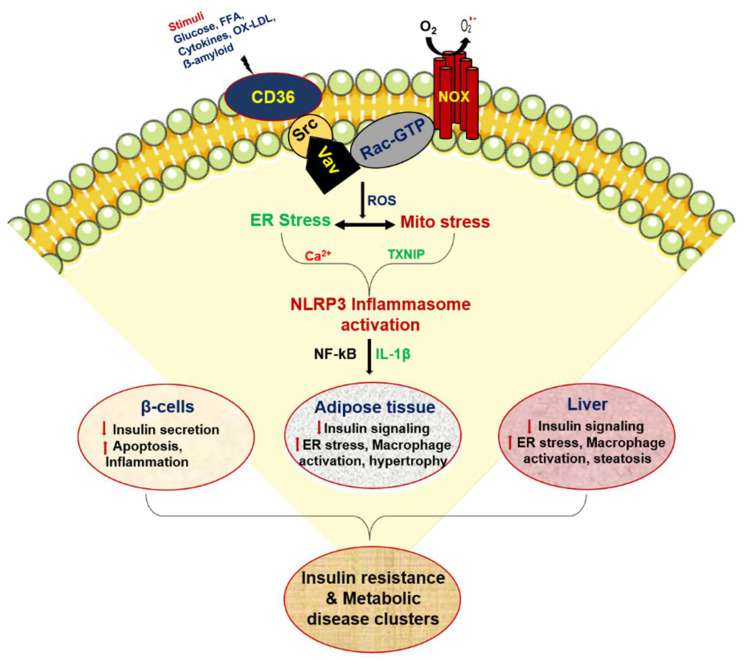
CD36 redox signaling mediates peripheral insulin resistance during metabolic disease.

In an unforeseen finding, CD36 signaling through lysosomal impairment was shown to promote inflammasome activation in adipose tissue from obese mice, suggesting that lysosomes may be vital in obesity-induced adipose tissue inflammation [162]. In addition to these proposed mechanisms, the central part of inflammasomes is specifically activated through lysosome-dependent pathways in macrophages and adipose tissue from obese mice [163,164]. This signaling pathway is subordinate to the activation of the CD36-FYN kinase-mediated Tyr353 phosphorylation of IP3R1, which leads to IP3R1-mediated Ca^2+^ exchange from the ER to the lysosome, and this causes lysosomal impairment and inflammation in adipocytes. The inhibition of CD36 palmitoylation reduces both FYN kinase activation and Tyr353 phosphorylation of IP3R1, which may contribute to reduced lysosomal Ca^2+^ overload and inflammation in adipocytes. A recent study by Wang et al. demonstrated that palmitoyl acyltransferases DHHC4 and DHHC5 control the palmitoylation of CD36 in directing fatty acid uptake in adipose tissues [165]. In addition to adipocytes, CD36 palmitoylation and the localization of CD36 on the plasma membrane of hepatocytes are significantly increased in patients with non-alcoholic steatohepatitis (NASH), as well as in livers from mice with NASH. The inhibition of the palmitoylation of CD36 protects mice from NASH and the inflammatory response [166]. In addition, membrane expression of CD36 was shown to be elevated in livers from mice and humans with non-alcoholic fatty liver disease during the aging process [167]. In support of this, a recent study by Chong et al. demonstrated that sustained exposure to amyloid-beta (Ab) drives senescence-associated secretory phenotype (SASP) via pro-inflammatory cytokine production and cell-cycle arrest in both epithelial cells and fibroblasts at the CD36–NF-κB signaling axis [168]. Therefore, CD36 activation has been proposed as a novel inflammatory agent to target and promote healthy aging and lifespan extension (Figure 3). Further research is required to identify CD36 targets in inflammatory aging disease. Based on its role in FFA uptake and the inflammatory process, it has been suggested that CD36 is involved in cardiovascular disease [169,170,171]. The pathophysiological role of CD36 in the cardiovascular system is discussed in the following recent reviews [172,173,174,175]. 

## 6. Conclusions

Experimental and clinical studies have fortified the noteworthiness of CD36 in metabolic disorders. CD36 has shown to act as a gatekeeper of various intracellular signaling systems that induce cell dysfunction under an excessive nutrition milieu. CD36 initiating redox signaling revealed the core pathophysiology of metabolic disorders such as diabetes, and promising therapeutic targets are expected. However, several questions regarding its impact on metabolic disease progression will need to be answered in future studies. Understanding this complex situation and the therapeutic agents that modulate the CD36 function could have a considerable impact on the treatment of metabolic diseases such as insulin resistance and diabetes.
